# Understanding the role of natural and anthropogenic forcings in structuring the periphytic algal assemblages in a regulated river ecosystem

**DOI:** 10.1038/s41598-023-27773-3

**Published:** 2023-02-02

**Authors:** Mohd Sharjeel Sofi, Aadil Hamid, Sami Ullah Bhat, Irfan Rashid, Jagdish Chandra Kuniyal

**Affiliations:** 1 Aquatic Ecology Lab, Department of Environmental Science, University of Kashmir, Srinagar, 190 006 India; 2 Department of Botany, University of Kashmir, Srinagar, 190 006 India; 3 Govind Ballabh Pant National Institute of Himalayan Environment (NIHE), Kosi-Katarmal, Almora, Uttarakhand 263 643 India

**Keywords:** Ecology, Environmental sciences

## Abstract

Periphytic algal assemblages in the River Sindh of Kashmir Himalaya were studied in relation to environmental factors and anthropogenic alterations like flow regulation for Run-of-River hydropower plants to understand their ecology in a regulated river ecosystem. Sites were sampled from unregulated, regulated, and downstream reaches along the river on a seasonal basis from the year 2017 to 2019. A total of 48 species were identified, spread over 31 genera. Non-metric multidimensional analysis showed clear distinction in periphytic algal assemblage samples based on sites and potentially some more minor distinction based on seasons rendering the sites into two distinct groups (G1 and G2). The ADONIS test showed that the groups (G1 and G2 sites) do not significantly differ in terms of how communities differ from one another, but there is a difference in species compositions based on seasons. However, the betadisper test indicated that groups (G1 and G2 sites) and seasons present homogeneity among group dispersions (compositions vary similarly) while having significantly different compositions. Geo-physical factors (discharge and altitude) accounted for most variations, while the scraper community played a minor role. This study provides scientific insights related to the ecology of a regulated Himalayan river and may provide information relevant to managing the River Sindh sustainably.

## Introduction

Periphyton, “a biofilm matrix of algae and bacteria that grows on the benthic substrate” is a key primary producer and a crucial component of rivers' ecological functioning^1,2^, providing food for both invertebrates and vertebrates in the benthic food web^3^. Periphytic algae have great diversity and comprise prostrate, stalked, and filamentous forms^4,5^. These forms react differently to environmental factors and grazing^6^. Due to the critical role of periphyton in stream food webs and ecosystem function, it is critical to understand the processes affecting the composition and biomass of periphyton^7^. Stressors are known to induce alterations in the biological structure and functioning of river ecosystems^8,9^. Scientific research has traditionally concentrated on the effects of a single principle stressor on aquatic ecosystems^10^. However, there have been efforts to understand how several disturbances might influence ecosystems simultaneously since this represents more realistic conditions^11^. In river ecosystems, the natural flow regime is often considered the principle abiotic variable essential for sustaining the ecological integrity of the rivers^9,12,13^. River flow regimes have been altered to meet human demands for transportation, water supply, flood management, or hydroelectric power^14^. These alterations disrupt the natural connectivity and affect the downstream transport of carbon and nutrients^15^. The regulation of flow may affect the composition of periphyton and the abundance of a particular species^16^, resulting in an increase or decrease in their numbers depending on the characteristics of the sampling site and the precise nature of the hydrological alteration^17^. In biological communities, patterns in species composition are determined by both regional and spatial forces^18^. For instance, altitude and geographical location may significantly influence the presence, absence, and abundance of algal species^19^. Thus, to thoroughly examine the weir-induced serial discontinuity, it is necessary to relate the upstream and downstream sides of the weirs in terms of their abiotic and biotic habitat features and take into account all the main taxonomic groupings^20^.

Understanding the impact of habitat and flow changes is crucial for sustaining the biodiversity and ecosystem services of freshwater ecosystems^21,22^. The flow regime is a crucial factor determining the periphyton growth in gravel bed Rivers^23^. Studies have identified both positive and negative associations between water velocity and periphyton^24^. These different results are likely due to variations in the form of periphyton growth across the rivers. For example, dense, prostrate periphyton may expand with velocity because it gains from greater nutrient supply rates at higher velocities, but filamentous algae diminish with an increase in velocity as the scouring of filaments rises^25^. Englund and Malmqvist found that species richness was lower at sites with the low flow than at areas with natural flow regimes, this may be attributed to the low food supply for grazers at sites with reduced flow^26^. The velocity of the water may affect the foraging efficiency of grazers and their function in shaping algae communities. Grazer-periphyton interactions may be influenced by the velocity variations resulting from reduced discharge. At low velocities, the grazing caddisfly *Agapetus boulderensis* lowers periphyton biomass and also alters the algal community structure. However, at high velocities, they only decrease periphyton biomass^27^. Such influence of water velocity on the efficiency of grazing is species specific. For instance, *Glossosoma verdona*, a caddisfly, was found to be a more efficient grazer at higher velocities, but the grazing efficiency of mayflies, *Baetis bicaudatus,* and *Drunella grandis* did not substantially alter with velocities^28^. Water velocity also influences the behavioral responses of invertebrates to algal resources. The behavior of invertebrates in response to algal resources is also influenced by water velocity. When periphyton levels are low, *Helicopsyche borealis* drift increases; however, at high velocities, passive drift predominates and no correlation exists between drift and periphyton levels^29^.

Periphyton may be controlled by bottom-up and top-down factors^30–32^, and the nutrients and herbivory affect the degree of periphyton response in streams^7^. Benthic food-web research often describes the influence of enrichment (bottom-up) or herbivory (top-down) playing a major role in the benthic food webs^33^. Various experimental and field investigations have shown that macroinvertebrate scrappers (grazers) and snails may influence periphyton through top-down control^34^. Scrapers are a distinct form of functional feeding group of macroinvertebrates that feed on periphyton. Scrapers may therefore react to the shift in food resource availability. Numerous investigations have shown a substantial correlation between algal development and scraper herbivory. However, the link is complicated because it is not a one-way interaction. That is, both algae and benthic invertebrate scrapers affect each other^35,36^. The excretion of fecal pellets by grazers may impact nutrient bioavailability in the periphyton^37,38^ and/or disrupt the biofilm, allowing for increased nutrient delivery from the water column to the periphyton^39^. According to previous research, algal biomass typically falls in the presence of herbivory^35,40^. On the other hand, these patterns are highly dependent upon the type of algae and herbivores. In certain circumstances, herbivory on overstory algae may benefit the population of understory algae in periphyton by increasing habitat availability^40^. As a result, macroinvertebrate feeding might indirectly influence periphyton activity and growth. Thus, it is important to look at both environmental variables and herbivory effects together.

However, studying the spatial and geographical impacts on algal composition is a foundational element in characterizing ecological patterns, thus increasing the reliability of biomonitoring, which is important for the long-term management of rivers^41^. Studying river biota with abiotic factors and recognizing the spatial structure of biotic assemblages and their primary drivers has become an emerging priority in community ecology to shape conservation efforts at the regional and global levels^42,43^. Despite this, detailed information about the relationship between hydrological factors and benthic organisms remains elusive^44^, and no such study has been conducted in a regulated river ecosystem in Kashmir Himalaya. In this context, we hypothesized that periphytic algal assemblage composition and structure would be strongly associated with the altitude, discharge, and abundance of scrapers/ herbivory in the River Sindh—regulated by Run-of-River hydropower operations in Kashmir Himalaya.

## Materials and methods

### Study area

Kashmir valley is situated between geographical coordinates of 34° 6′–34° 27′ N and 74° 40′–75° 35′ E. The territory was shaped by tectonic processes and has a strong evolutionary connection to the northwestern Indian Himalayan region (IHR), which exerts a substantial impact on its geographic identity^45^. The region's Sindh Basin stretches between 34° 11 17 N to 34° 46ʹ 30ʹʹ N and 74° 5710 East to 75° 63ʹ 4ʹʹ East. The elevation of the Sindh basin ranges from 1563 to 5375 m. From the base of Saskut (4693 m.a.s.l.) in the Ogput Range, the Sindh river originates from the Panjtarni glacial fields located at an altitude of 4250 m.a.s.l^46,47^. The River Sindh has a length of around 116 km and a basin area of 1683.24 km^2^^48^. The River meanders through Sonamarg (a scenic tourist destination) located at a height of about 2730 m.a.s.l. Climate change is significantly affecting the basin, as seen by the shrinking size and volume of the Thajwas and Naranag glaciers. Human-induced interferences such as the construction of three hydropower projects have resulted in the river's fragmentation in some areas, which intensifies during the dry season. The current rate of urbanization jeopardizes long-term development, and an unchecked tourist influx and the conversion of the pastures to built-up and other proposed infrastructure should be avoided to preserve the ecology of the area^49^.

### Selection of sampling sites

**Figure 1:**
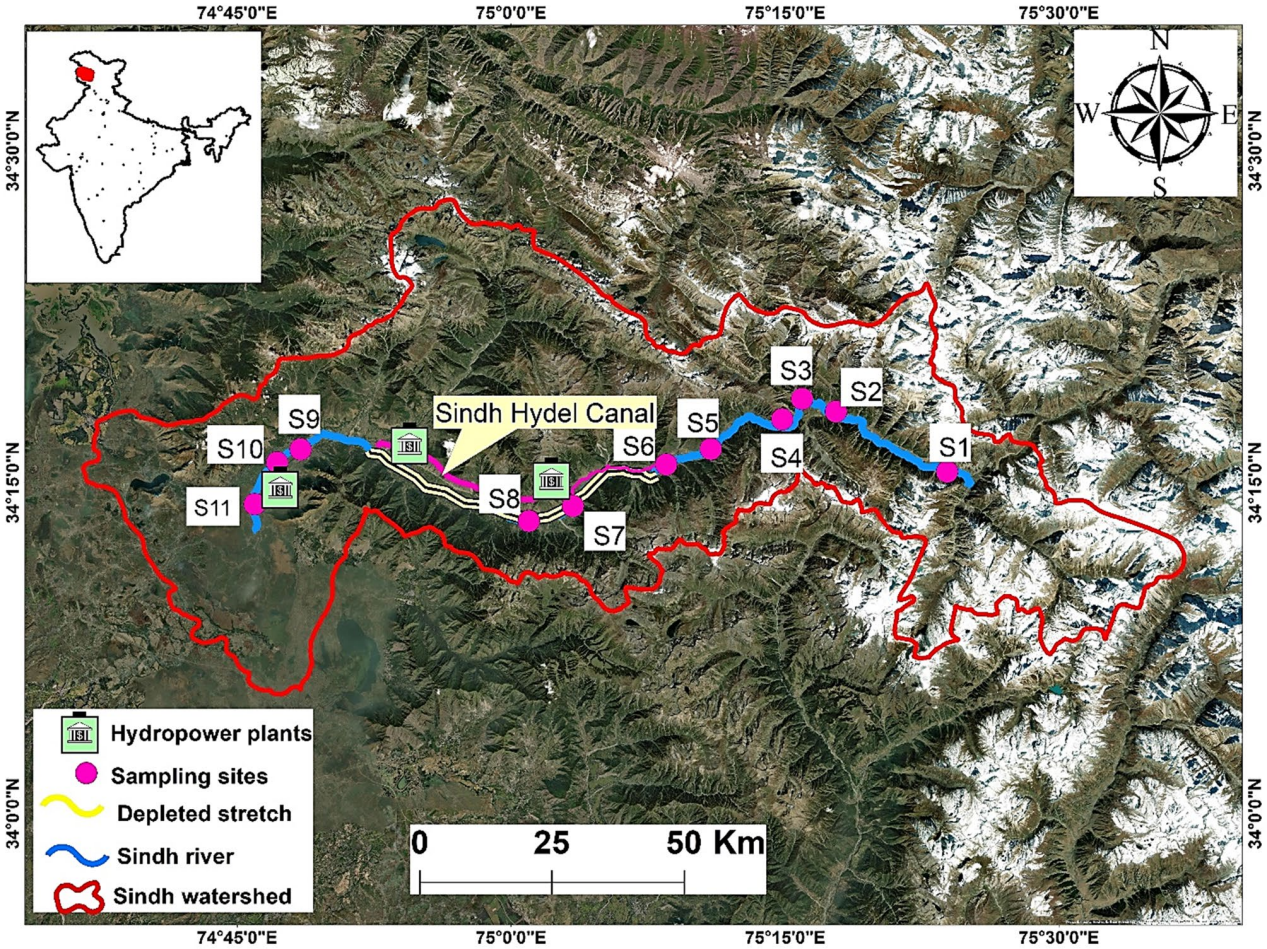
Study area showing the eleven sampling sites along the River Sindh.

The datasets regarding elevation, discharge, macroinvertebrates, and periphytic algae were collected from December 2017 to December 2019 on monthly basis in the Sindh basin. Eleven sampling stations (Table 1 in supplementary file) representing the River Sindh and its main tributaries were carefully selected (Fig. 1). Apart from accessibility, the sample locations were selected after careful evaluation of natural and human activities that may have an impact on periphytic algae. To address the lack of understanding regarding the effects of flow regulation on the structural pattern of periphyton and the grazer-periphyton interaction, samples were gathered from sites in the upper, depleted, and lower sections of the River Sindh (Fig. 1). Moreover, the upper sites from S1 to S6 are affected by tourism and Z- Morh Tunnel construction, whereas the sites S7 and S8 are present below the weirs of the Run-of-River hydropower plants. The lower section of the river host three sites (S9–S11) that are affected by sewage inflow, tourism activities, and sand mining.

#### Periphytic algae

Blades and brushes were used to collect samples having a surface area of 5 cm^2^ from each location. The scraped region was rinsed in a tray before being placed in a vial of appropriate volume^50^. 1 mL Lugol's iodine and three drops of 4 percent formalin were used to preserve the samples^51^ and then after, the samples were increased to a constant volume^62^ of 25 ml. Samples were identified using standard keys^52–55^. Samples were examined and enumerated using an inverted light microscope at 40× and 100× magnifications. Individual cells were counted and written down in a lab notebook.

#### Macroinvertebrates

Macroinvertebrates were collected from stream sediment by kicking and moving it with our hands and feet^56^. This approach dislodges benthic macroinvertebrates, which are subsequently collected using a D-net with a mesh size of 0.5 mm and a 30 cm broad base and 30 cm long^57,58^. D-net was placed approximately 0.5 m from the location on the streambed that needed to be disturbed, and the substrate was kicked for 1 min to dislodge the cobbles or gravel^59,60^. The method was repeated thrice in different places within a 10-m reach in the river to sample diverse velocity regimes and habitats until the 1 m^2^ region had been covered. Hand-collected samples were also taken from individual boulders in the water^61^. The samples were kept in 100% ethanol for long-term storage until the identification process was finished. Standard keys were used for the identification of macroinvertebrates^62–70^

#### Discharge and altitude

The area-velocity approach was used to determine the discharge. A flow probe was used to measure velocity automatically (Model FP111, Made in the USA). The area was determined manually by measuring the depth along a cross-section of the river at regular intervals of 5 m^71^. The coordinates and altitude of the sampling sites were determined using a handheld GPS (Montana 650, Garmin).

### Data analysis

Complex spatio-temporal dynamics in periphytic algae communities under-regulated and natural flow regimes, and their interactions with different environmental parameters were investigated using multivariate statistical approaches. We used the vegan package^72^ to carry out non-metric multidimensional scaling (NMDS), which is a good way to organize sites based on their similarities or dissimilarities. The NMDS uses Bray–Curtis dissimilarity to describe the pairwise dissimilarity among the sites in the smallest number of dimensions available^73,74^. In terms of biological assemblages, sites that are more similar to one another are ordinated together. After the NMDS, an analysis of similarity (ANOSIM) was used to see if there were differences between sites and seasons that were statistically significant. The packages "betapart" and "vegan" were used to compare and measure beta diversity using abundance data to understand the differences in the composition of species across groups and seasons. The Adonis test was then performed using the EcolUtils R package's "adonis. pair" function^75^. All tests were run with 999 permutations of statistical significance^76^. To investigate the degree of dissimilarity, caused by differences in the community composition and abundance of taxa^82^, we utilized the Bray–Curtis distance based on abundance data as a performance parameter for dissimilarity by using the "vegdist" function in R^77^. Further, to explore the periphytic algae community characteristics and their interaction with one another and environment parameters, Alpha diversity (Shannon’ H’, Species Richness, Chao1, and Pielou’s Evenness), Species richness, and Chao index^78^ were taken into account during the analysis. The analysis of variance (ANOVA) was performed to examine the variance in ecological characteristics of the periphytic algal assemblages between groups and seasons. Using the packages "viridis," "cowplot," "indicspecies," and "reshape2," we used indicator species analysis based on relative abundance (percent) to estimate the role of the most influential species to Bray–Curtis Dissimilarity between each pair of groups and seasons.

Finally, distance-based redundancy analysis (dbRDA) was performed to analyze the association between periphytic algal assemblages and environmental parameters across time using the packages "stringr," "dplyr," "tidyr," "vegan," and "ggplot2". Environmental variables were chosen for analysis using a forward selection approach (p 0.05) based on 9999 permutations. Before dbRDA, strongly correlated (Pearson's r > 0.80) environmental variables (multicollinearity) were eliminated, while the remaining variables were modified (z-transformation) to satisfy the premise of normality^79,80^. Because of their unbiased character, the adjusted determination coefficients for environmental variables derived from unrestricted and restricted ordination were included in the analysis^80^. Environmental factors involved in determining the pattern of periphytic algal assemblages were then identified. R Software Version 3.4.0 was used to perform a statistical analysis of the data^81^.

## Results and discussion

### Periphytic algae patterns

**Figure 2:**
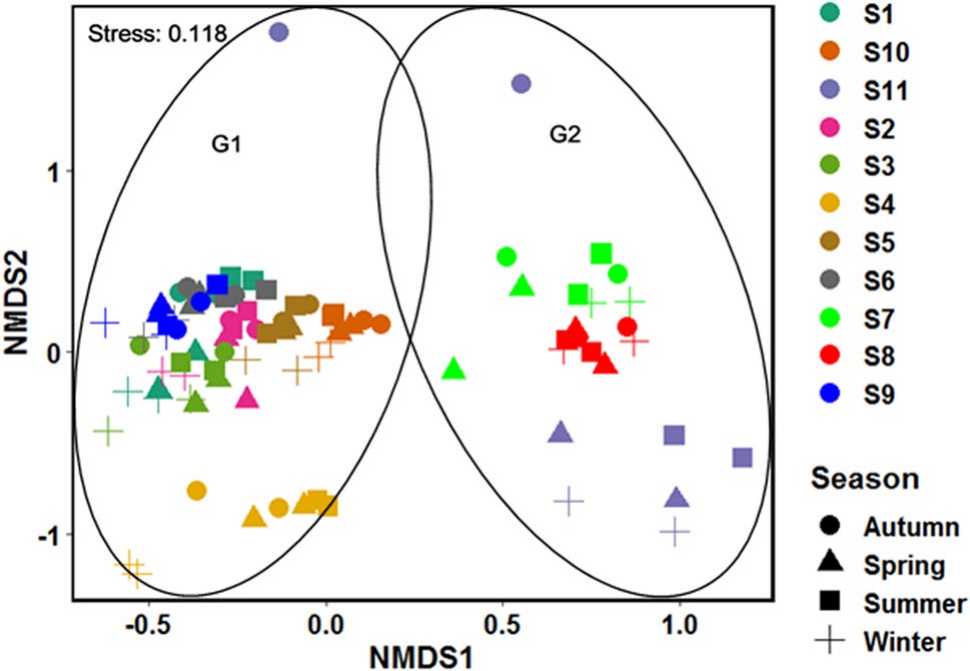
NMDS ordinates of periphytic algal abundance data at eleven sampling stations across the seasons.

NMDS output indicates that there was a quite distinction in datasets based on sites and potentially also some distinction based on seasons. NMDS analysis of periphytic algae assemblages (count) data, followed by ANOSIM analysis, indicated a significant differentiation across locations (R = 0.6555, p = 1e−04). However, there was a modest but statistically significant seasonal difference in periphytic algae assemblages (R = 0.07057, p = 0.0015), albeit to a lower degree when statistical significance is calculated using 9999 permutations (Fig. 2). An R-value around one (1) suggests that the groups are dissimilar, while an R-value near zero (0) implies that there is little difference inside and between the groups. NMDS statistical techniques rendered sites into distinct groups G1 (including sites S1, S2, S3, S4, S5, S6, S9, and S10) and G2 (S7, S8, and S11) based on periphytic algae abundance (count) data (Fig. 2). This reveal the role of flow alteration in configuring riverine algae assemblages^44^ and macroinvertebrate scrapper abundance. The grouping of the sites may also be attributed to the low current velocity at G2 sites (having an avg. discharge of 25.66 (m^3^/s) at site S7 and S8 (regulated sites), as against to the G1 sites having avg. discharge of 30.84 (m^3^/s). The flow regulation enhances the deposition of fine sediments and negatively affects the benthic communities^82^, by reducing their density and diversity^83,84^. Similarly, flow regulation at S7 and S8 may have influenced the periphyton abundance^16^, as a result, their numbers vary according to on-site characteristics and the exact form of hydrological alteration. The flow velocity is deemed to be the most important element influencing the growth of aquatic biota, especially periphyton^85^. The distinct outliers formed during the autumn season for S11 may be due to the low density and diversity (only 4 species reported in the autumn season) of the periphyton. This could be because dredging and sand mining substantially reduce the diversity and density of benthic organisms^86^. Sediments have a variety of consequences on periphyton, including light attenuation^82^, abrasion, a reduction in the amount of hard substrate accessible for colonists, and a loss in hydraulic connection with the hyporheos^87^. Thus, in our study, sites falling under the G2 group were grouped due to the impacts of flow regulation and a similar pattern of periphytic algal abundance.

### Contribution of periphytic algae as indicator taxa

**Figure 3:**
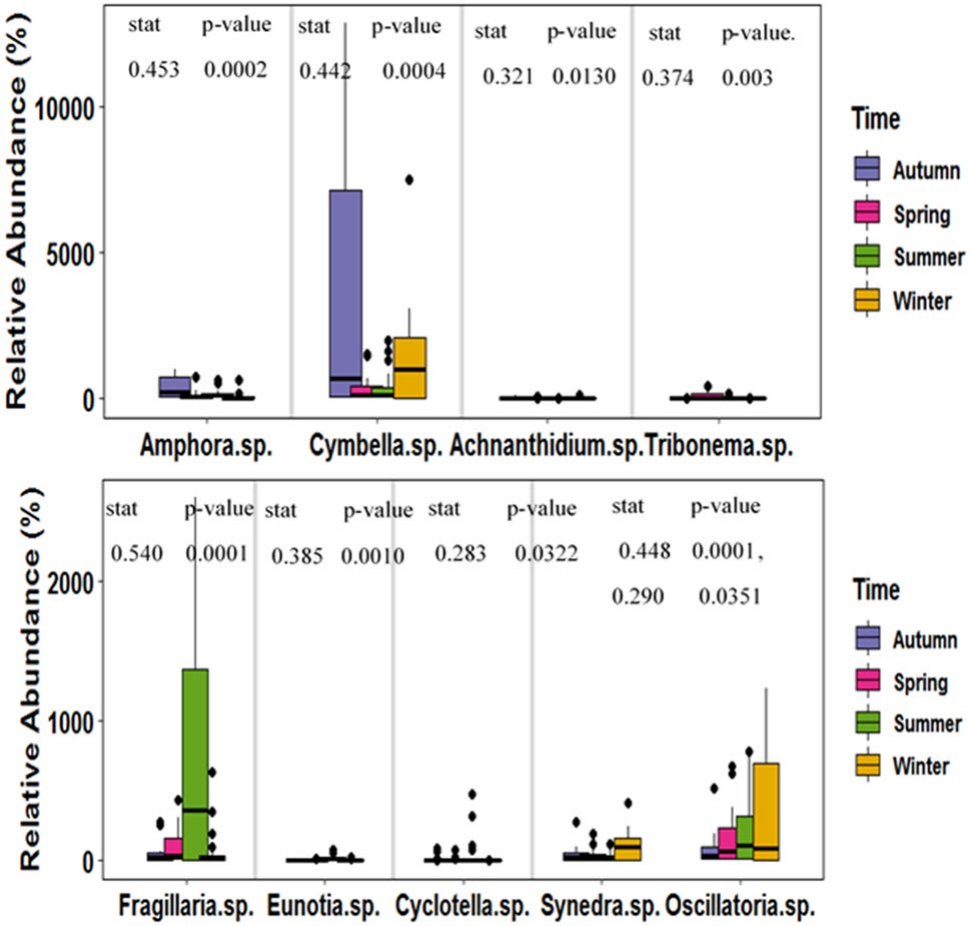
Relative abundance of occurrence of each taxa in different seasons.

**Figure 4:**
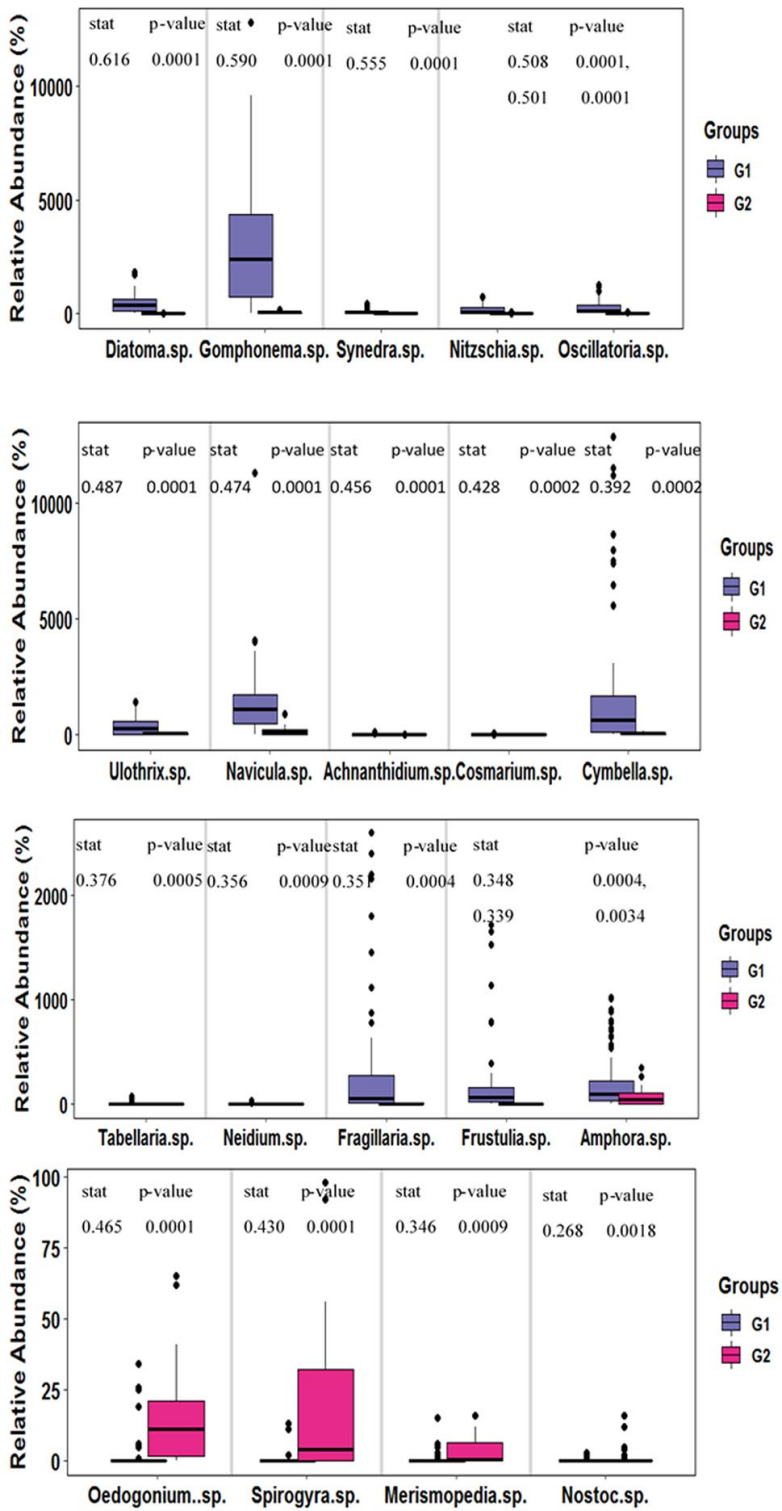
Showing group-based (cluster of sites) relative abundance of each taxa.

The indicator species analysis based relative abundance of each taxon across groups and seasons was investigated independently at a significance level of p < 0.05 (Figs. 3 and 4). The indicator species analysis results revealed that *Diatoma* sp., *Gomphonema* sp., *Synedra* sp., *Nitzschia* sp., *Oscillatoria* sp. *Ulothrix* sp. *Navicula* sp., *Achnanthidium* sp., *Cosmarium* sp., *Cymbella* sp., *Tabellaria* sp., *Neidium* sp., *Fragillaria* sp., *Frustulia* sp., and *Amphora* sp. were characteristic of G1 while *Oedogonium* sp., *Merismopedia* sp., *Spirogyra* sp., and *Nostoc* sp. were characteristic of G2. The dominance of the Bacillariophyceae in the River Sindh indicates that the pattern of the periphytic algal assemblages followed the same trend as has been the case in the majority of the world’s rivers^88,89^. Throughout the research period, diatoms dominated the vegetation, while the species composition at regulated and one downstream site altered. The dominance of the species belonging to the Bacillariophyceae at the G1 sites may be due to the presence of a good concentration of SiO_2_ in the river, which aids them in the frustule formation^90^ and demonstrates their ability to flourish in cold waters. The abundance of Bacillariophyceae at these unregulated sites shows their oligotrophic nature^91^. Species within the genera *Gomphonema* sp. and *Frustulia* sp. were characteristic of upstream and unregulated river reaches^92^. The G2 sites include two regulated sites (S7 and S8) and the flow variation at these sites is minimal and remains constant for weeks or months^93^ which explains the domination by blue-green algae (Cyanobacteria), particularly colonies of *Nostoc* sp.^94^ and filamentous green algae *Oedogonium* sp. which indicates the nutrient enrichment of these sites (Fig. 4). Green algal dominance in the G2 sites is most likely related to higher water temperatures and consistent low flows. Filamentous green algae densely grow in regulated rivers, which can be corroborated by our findings as well^95^. Cyanobacteria flourished due to low flow, favorable physicochemical conditions, and suitable sediment size^39^. Based on seasons, *Amphora* sp., *Cymbella* sp., and *Achnanthidium* sp. had a greater preference for Autumn, *Tribonema* sp., preferred Spring, *Fragillaria* sp., *Eunotia* sp., and *Cyclotella* sp., for Summer, and *Synedra* sp., for Winter seasons (Fig. 3). Among the numerous genera of Bacillariophyceae, *Synedra* sp. exhibited the greatest frequency and productivity in the winter^96^. The relatively high growth of periphytic algal assemblages in the autumn season may be due to the low flow (6.1 m^3^/s), which reduces the scouring action of the water and more light reaches the bottom, which enhances the growth of the algae^68^. The relative decrease in the periphytic algal assemblage densities observed in the summer may be attributed to the increase in the discharge which reaches the maximum of 78.9 m^3^/s. The increase in discharge enhances the scouring process from the substrate^97^. The reduction in the density of the periphytic algal assemblages in the winter season may be attributed to the fall in the temperature of the water and the loosening of periphyton from the substrate^25^. Although it is often assumed that periphyton standing stocks are substantially smaller in the winter than in the summer^16^, late-autumn to winter maxima have also been reported^98^. Our results corroborate the perception that periphyton standing crop decreases during winters due to low irradiance^99^ and low temperature^100^.

### Measuring and comparing beta-diversity

**Figure 5:**
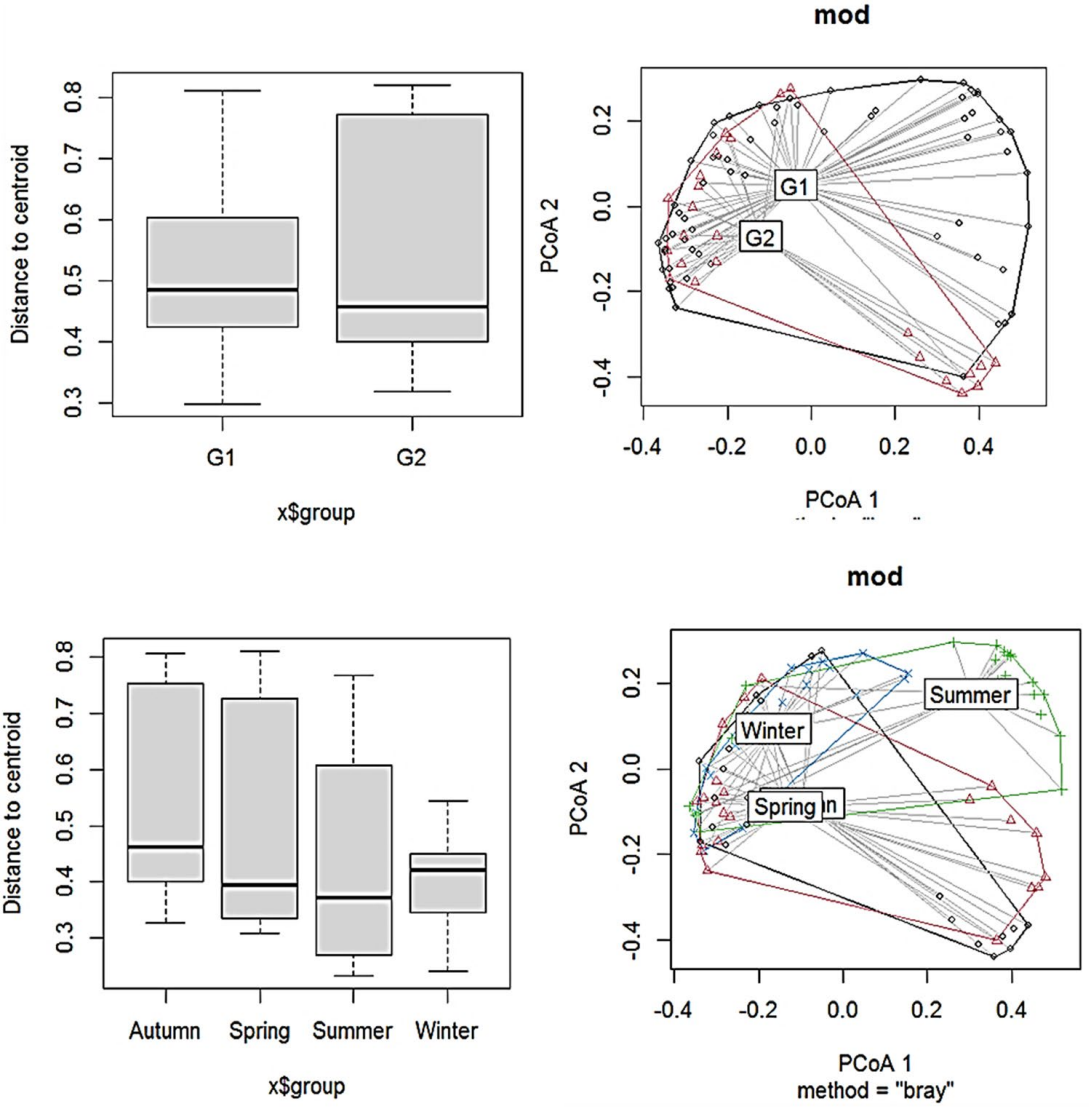
Betadisper plot showing the difference in species compositions across seasons and sites.

The betadisper test indicated that groups (G1 and G2 sites) and seasons present homogeneity among group dispersions (compositions vary similarly) while having significantly different compositions (Fig. 5). ANOVA treatments showed that (G1 and G2 sites) do not differ significantly in how communities vary from each other (F = 0.483, p = 0.4889). However, the ADONIS test results indicated that our groups (G1 and G2) do not significantly differ in terms of how communities differ from one another, but that there is a difference in species compositions based on seasons (F = 4.1048, R^2^ = 0.0456, p = 0.002). This suggests the weir alters the structure, productivity, and diversity of the periphytic algal assemblages in the stream ecosystems^9^. This may be due to the similarities observed among the sites falling under the G1 and G2 groups having similar hydrological, and physicochemical variables and degrees of flow regulation^39^. The betadisper test plot indicates that seasons present homogeneity among group dispersions (compositions vary similarly) while having significantly different compositions. ANOVA treatments showed that seasons did differ significantly in how communities vary from each other (F = 3.1038, p = 0.0308). ADONIS test results indicated that there is a difference in species compositions across seasons (F = 6.0542, R^2^ = 0.17776 p = 0.001). Seasonality has been shown to significantly impact periphyton community composition in many studies^101–103^. On similar lines, the community composition of periphyton also demonstrated a seasonal fluctuation in our investigation. For instance, the lower density and change in composition in the winter season may be attributed to a decrease in solar energy and temperatures in temperate latitudes, which impede the organism's physiology and may cause ice formation, affecting in-stream conditions and potentially affecting stream species and populations^104^.

### Analysis of variance (ANOVA)

**Figure 6:**
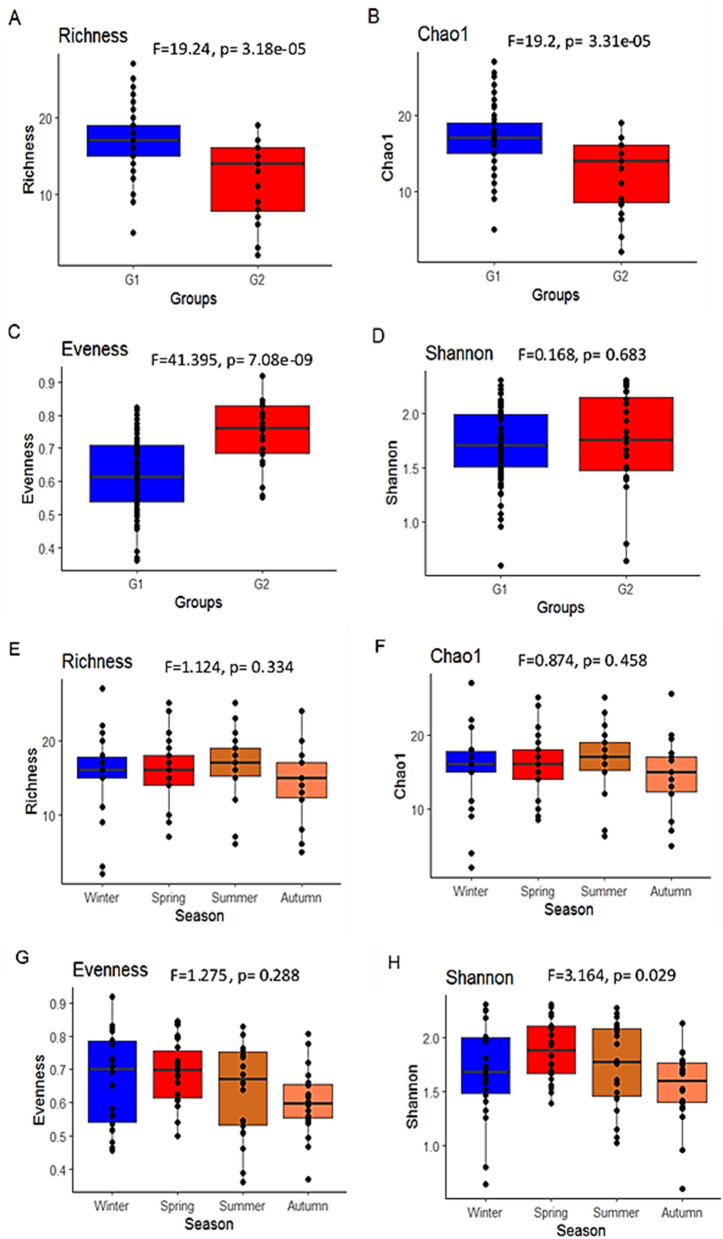
Showing variation in species richness, Chao1, and Evenness metrics among seasons and groups.

The one-way ANOVA revealed a statistically significant difference in species richness, Chao1, and Evenness metrics among G1 and G2 sites (p < 0.05). However, there is no statistically significant difference in species richness, Chao1, and Evenness among the seasons (Fig. 6). Shannon index displayed statistically significant variation among seasons and statistically insignificant variation among groups.

### Distance-based redundancy analysis (dbRDA)

**Figure 7:**
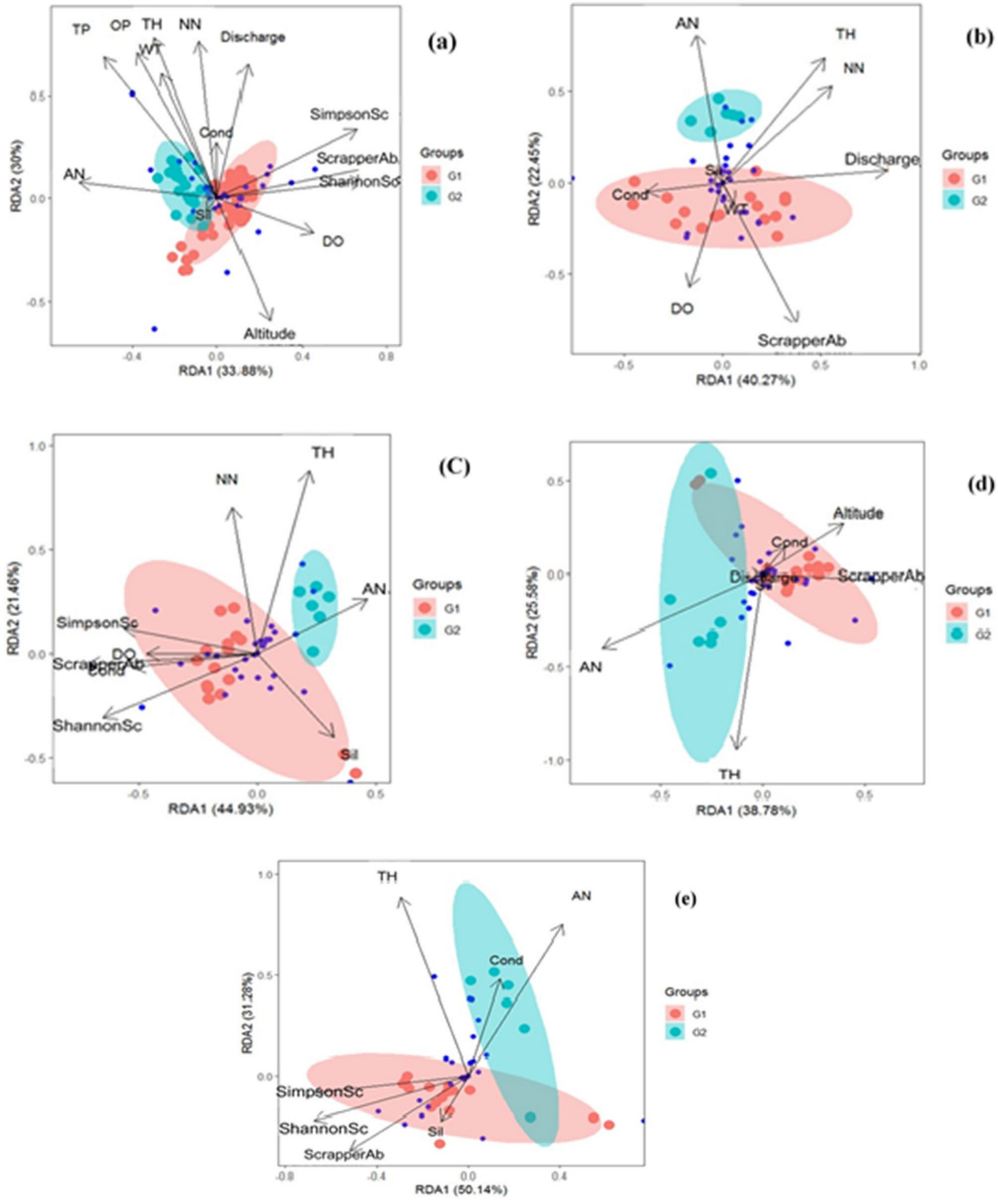
Distance-based redundancy analysis (dbRDA) plot showing the variance in periphytic algal assemblages explained by environmental factors (**a**) Group-wise (**b**–**f**) Seasonal wise.

In lotic systems, the composition and distribution patterns of stream periphytic algal assemblages are governed solely or in concert by a collection of environmental variables and anthropogenic interventions functioning on several scale dimensions^105^. This study describes variability among the periphytic algae as more related to discharge, altitude, ShannonSc (Shannon Index of Scrappers), SimpsonSc (Simpson Index of Scrappers), ScrapperAb (Abundance of scrappers), AN (Ammonical-nitrogen), NN (Nitrate-nitrogen), TH (Total Hardness), and DO (Dissolved Oxygen). Based on spatial scale, Total Phosphorus (TP), Ortho-phosphorus (OP), Total Hardness (TH), Nitrate–Nitrogen (NN), Water Temperature (WT), Ammoniacal Nitrogen (AN), discharge, altitude, SimpsonSc, ShannonSc, and ScrapperAb significantly contributed to the variability of periphytic algal assemblage composition and structure with RDA1 (Permutation test for axes, F = 20.86, p = 0.001) explaining 33.88% and RDA2 (Permutation test for axes, F = 18.46, p = 0.001) explaining 30% variation respectively. Permutation test for rda under reduced model at 999 permutations showed that the model is significant (F = 4.48, p = 0.001) (Fig. 7a). In summers, TH, AN, NN, discharge, conductivity, silica, ScrapperAb, ShannonSc, and SimpsonSc significantly contributed to the variability of periphytic algal composition and structure with RDA1 (Permutation test for axes, F = 13.11, p = 0.001) explaining 44.93% and RDA2 (Permutation test for axes, F = 6.24, p = 0.001) explaining 21.46% variation respectively. Permutation test for rda under reduced model at 999 permutations showed that the model is significant (F = 3.324, p = 0.001) (Fig. 7b). In the spring season, DO, discharge, NN, TH, AN, and ScrapperAb significantly contributed to the variability of periphytic algal composition and structure with RDA1 (Permutation test for axes, F = 9.314, p = 0.001) explaining 40.27% and RDA2 (Permutation test for axes, F = 5.19, p = 0.001) explaining 22.45% variation respectively. Permutation test for rda under reduced model at 999 permutations showed that the model is significant (F = 2.569, p = 0.001) (Fig. 7c). During autumn, AN, TH, altitude, and ScrapperAb significantly contributed to the variability of periphytic algal composition and structure with RDA1 (Permutation test for axes, F = 5.95, p = 0.001) explaining 38.78% and RDA2 (Permutation test for axes, F = 3.29, p = 0.083) explaining 25.58% variation respectively. Permutation test for RDA under reduced model at 999 permutations showed that the model is significant (F = 2.19, p = 0.002) (Fig. 7d). During winter, TH, AN, SimpsonSc, ShannonSc, and ScrapperAb significantly contributed to the variability of periphytic algae composition and structure with RDA1 (Permutation test for axes, F = 11.49, p = 0.001) explaining 50.14% and RDA2 (Permutation test for axes, F = 7.16, p = 0.015) explaining 31.28% variation respectively. Permutation test for rda under reduced model at 999 permutations showed that the model is significant (F = 3.27, p = 0.001) (Fig. 7e). Redundancy analysis results revealed the significant contribution of altitude, discharge, AN, TH, DO, TP, OP, DO, ShannonSc, SimpsonSc, and ScrapperAb in driving the periphytic algal composition and structure summer, spring and winter seasons. However, in the autumn season, only altitude, TH, AN, and ScrapperAb are the only significant factors. These observations could be attributed to the flow regulation which affects the structure and function of benthic algal communities. Moreover, we must take into account that changes in flow regime by the operation of Run-of-River hydropower plants may have varying degrees of impact on the physicochemical characteristics of water in the bypassed reaches of the river^106^. As a result, the combination of stressors might be a substantial factor in determining the variations in the periphytic algal assemblages. Grazing plays a key role in stable ecosystems, and it may have a big impact on the result of perturbation and resource supply interactions on species membership^107^. The significant influence of ShannonSc, SimpsonSc, and ScrapperAb indices at G1 sites shows the top-down effects of herbivores on periphyton^108^. Scrappers' ability to reduce periphyton biomass is highly reliant on the interaction between their capacity to remove periphyton (which is partially determined by the number and kind of herbivores present) and the rate of periphyton growth. However, in the current study, the quantification of the role of the herbivory couldn’t be determined and must be a part of future investigations.

## Conclusion

The analysis of the data revealed that flow regulation by Run-of-River hydropower projects, altitude, and scraper abundance affect the structure and composition of periphytic algae. Furthermore, we found that changes in different environmental and biological parameters have different effects on the physicochemical parameters of water and benthic algal assemblages, even though they often have substantial cumulative impacts. As a result, the combination of many stressors plays a major role in structuring the periphytic algal assemblages. Our findings demonstrated that benthic algal assemblages have a clear relationship with the flow regime. Thus, this relationship has the potential to act as a good criterion for long-term biomonitoring programs as well as river management and policy recommendations. It is difficult to unravel and quantify the causal relationship between flow regime and ecological structure/function, particularly when using conventional taxonomic composition. In view of overcoming this problem, aquatic ecology needs to move into a manipulative or experimental analysis of periphytic algae to establish the role of environmental gradients in determining the community composition at a higher resolution.

## Supplementary Information

Supplementary Information.

## Data Availability

The data used/or analyzed that supports the findings of this study is available in the main manuscript file and from the corresponding author on reasonable request.
