# Supplementary material for: Understanding the role of natural and anthropogenic forcings in structuring the periphytic algal assemblages in a regulated river ecosystem
Source: Sci Rep. 2023 Feb 2;13:1882. doi: 10.1038/s41598-023-27773-3 (PMC9894984; doi:10.1038/s41598-023-27773-3)
Supplement: Supplementary file 1 — Supplementary Information. [file 41598_2023_27773_MOESM1_ESM.docx]

**Table 1. Coordinates and codes of the Sampling Sites**

| S. No | Name of the site | Code | Altitude(m) | Latitude | Longitude |
| --- | --- | --- | --- | --- | --- |
| 1 | Baltal | S1 | 2867 | 34^°^14ʹ39.70ʺ N | 75°15ʹ32.46ʺE |
| 2 | Sonamarg | S2 | 2812 | 34^°^18ʹ07.84ʺ N | 75°15ʹ47.99ʺE |
| 3 | Thajwas | S3 | 2798 | 34^°^17ʹ25.6ʺ N | 74°16ʹ10.55ʺE |
| 4 | Shutkari | S4 | 2647 | 34^°^18ʹ47.0ʺ N | 75°15ʹ56.00ʺE |
| 5 | Gagangir | S5 | 2275 | 34^°^16ʹ31.2ʺ N | 75°10ʹ56.20ʺE |
| 6 | Kulan | S6 | 1972 | 34^°^16ʹ04.8ʺ N | 75°09ʹ20.48ʺE |
| 7 | Sumbal | S7 | 1881 | 34^°^13ʹ56.63ʺ N | 75°03ʹ22.92ʺE |
| 8 | Ganiwan | S8 | 1801 | 34^°^13ʹ16.31ʺ N | 75°00ʹ57.51ʺE |
| 9 | Preng | S9 | 1731 | 34^°^16ʹ30.5ʺ N | 74°48ʹ28.03ʺE |
| 10 | Wail | S10 | 1663 | 34^°^16ʹ54.05ʺ N | 74°48ʹ10.3ʺE |
| 11 | Ganderbal | S11 | 1585 | 34^°^13ʹ24.34ʺ N | 74°46ʹ6.36ʺE |
